# Supplementary material for: Portable FRET-Based Biosensor Device for On-Site Lead Detection
Source: Biosensors (Basel). 2022 Mar 2;12(3):157. doi: 10.3390/bios12030157 (PMC8946079; doi:10.3390/bios12030157)
Supplement: Supplementary file 1 [file biosensors-12-00157-s001.zip › biosensors-1592399-supplementary/Supplementary Materials.pdf]

biosensors

Article

Portable FRET-Based Biosensor Device for On-Site Lead Detection

Wei-Qun Lai [1,2], Yu-Fen Chang [3], Fang-Ning Chou [1] and De-Ming Yang [1,2,*]

[1] Microscopy Service Laboratory, Basic Research Division, Department of Medical Research, Taipei Veterans General Hospital, Taipei 11217, Taiwan; dmyang@vghtpe.gov.tw
[2] Institute of Biophotonics, School of Biomedical Science and Engineering National Yang Ming Chiao Tung University, Taipei 11221, Taiwan; yang.deming2021@nycu.edu.tw
[3] LumiSTAR Biotechnology, Inc., Taipei City 115, Taiwan; yu-fen.chang@lumistar.com.tw
* Correspondence: dmyang@vghtpe.gov.tw

Citation: Lai, W.-Q.; Chang, Y.-F.; Chou, F.-N.; Yang, D.-M. Portable FRET-Based Biosensor Device for On-Site Lead Detection. *Biosensors* **2022**, *12*, 157. https://doi.org/10.3390/bios12030157

Received: 26 January 2022
Accepted: 28 February 2022
Published: 2 March 2022

Publisher's Note: MDPI stays neutral with regard to jurisdictional claims in published maps and institutional affiliations.

Copyright: © 2022 by the authors. Licensee MDPI, Basel, Switzerland. This article is an open access article distributed under the terms and conditions of the Creative Commons Attribution (CC BY) license (https://creativecommons.org/licenses/by/4.0/).

Additional Figures.

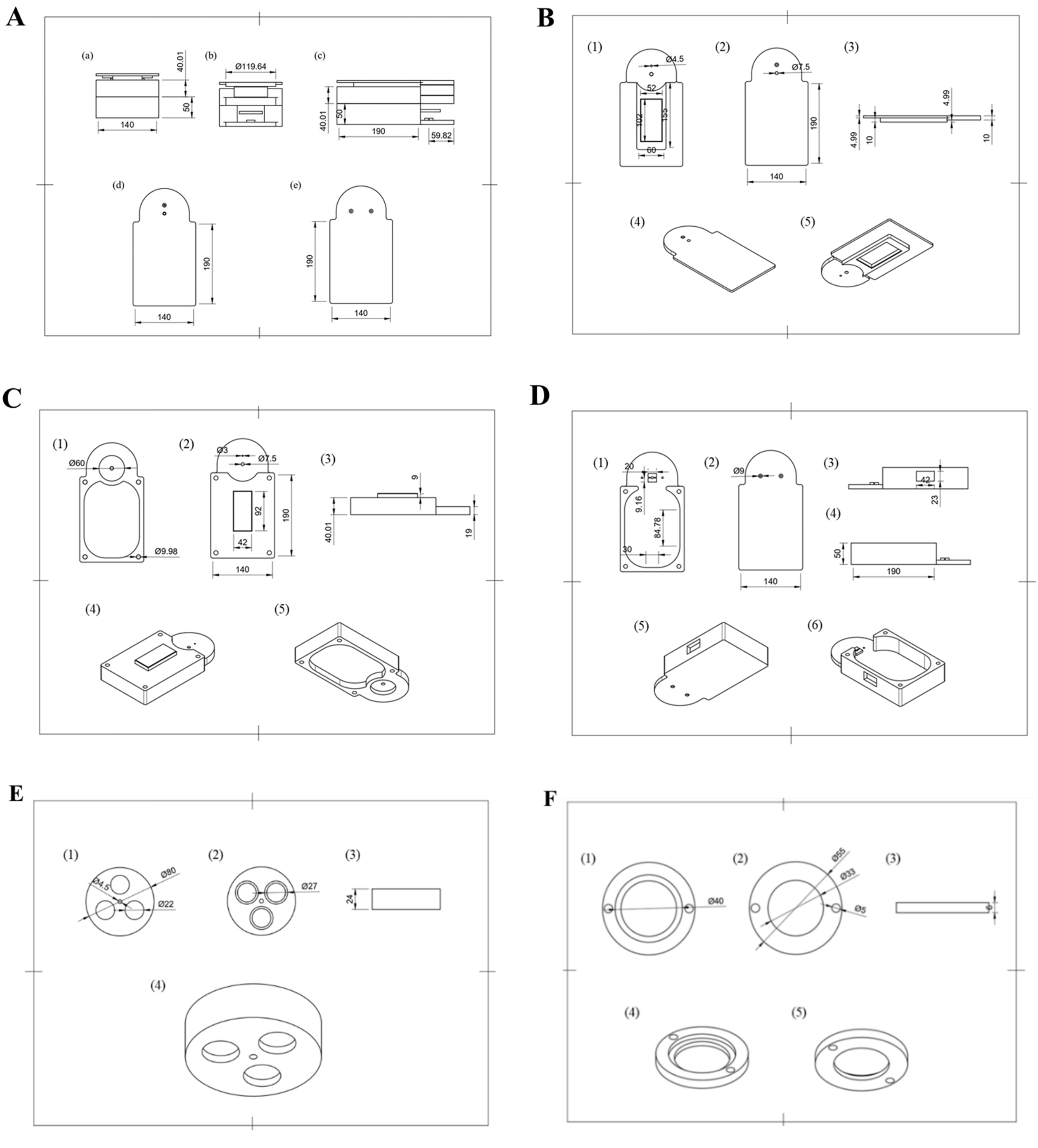

Figure S1. Design of the smartphone-based Pb sensing device in detail. (**A**) Outline structure design. (**B**) Top part design. (**C**) Middle part design. (**D**) Bottom part design. (**E**) Filter turret design. (**F**) Sample stage design.

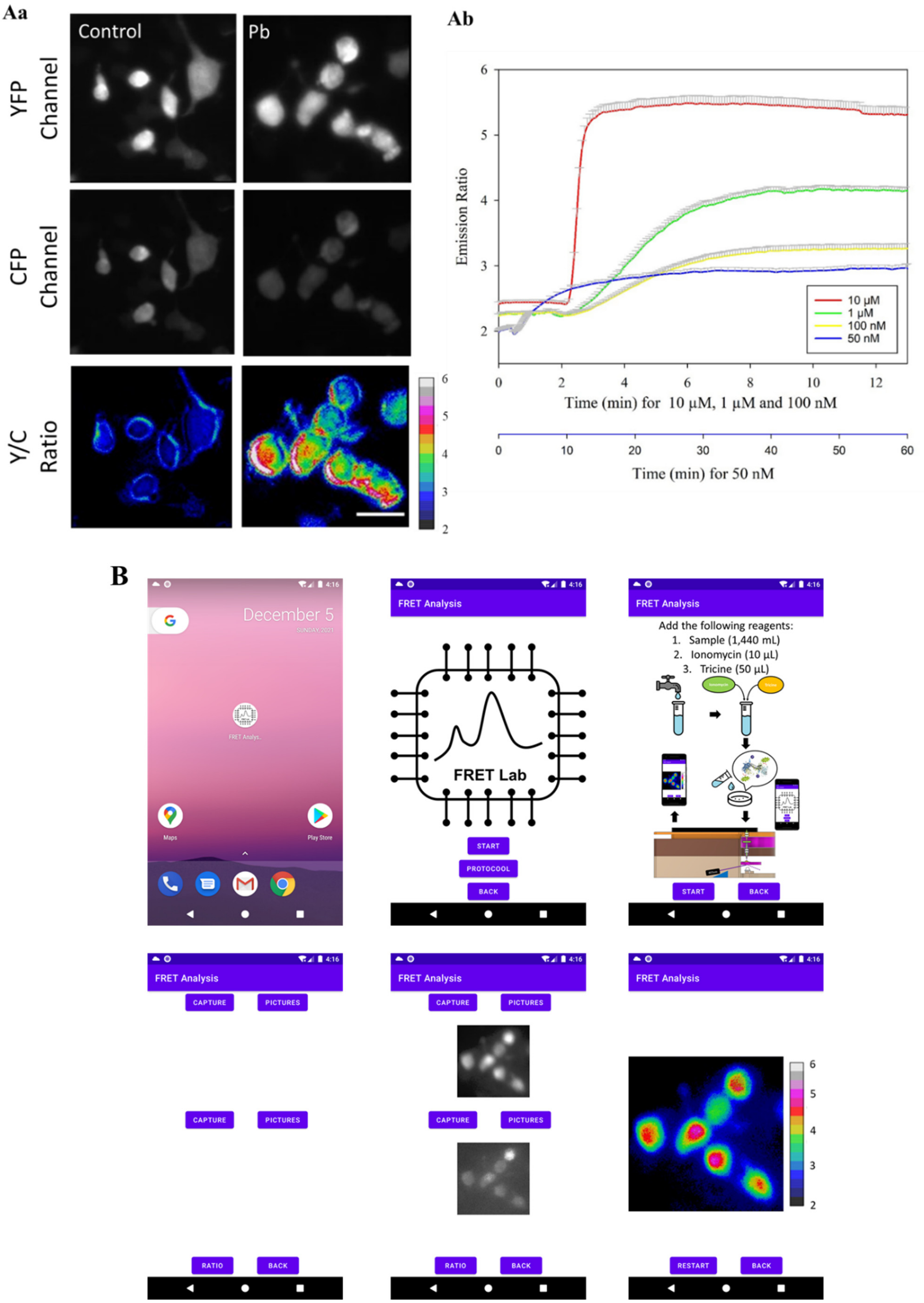

Aa
Control
Pb
YFP Channel
CFP Channel
Y/C Ratio
Ab
Emission Ratio
Time (min) for 10 µM, 1 µM and 100 nM
Time (min) for 50 nM
10 µM
1 µM
100 nM
50 nM
B
December 5
FRET Analysis
FRET Lab
START
PROTOCOOL
BACK
Add the following reagents:
1. Sample (1,440 mL)
2. Ionomycin (10 µL)
3. Tricine (50 µL)
CAPTURE
PICTURES
RATIO
RESTART

Figure S2. Sensing function check of Met-lead biochip through conventional FRET microscope and the software for pMet-lead. (**A**) Functional checking of Met-lead 1.44 M1 under a general FRET ratio microscope with 10 x objectives, representative images are shown in (**Aa**) (Control without Pb and Pb with 100 nM or Pb). Various concentrations of Pb (from 50, 100 man, to 1, 10 μM) were used under a conventional FRET imaging and shown in (**Ab**). (**B**) The flowchart of the iMet-lead app for smartphones integrated with the device pMet-lead. Android Studio was used through the Java language as an integrated development environment to construct the Android App, iMet-lead. To help users easily proceed Pb sensing with pMet-lead, iMet-lead can preview the operation procedure before sensing analysis. At the stage of preview and the following data acquisition, images of YFP and CFP will be seen on the screen of the smartphone under the interface of iMet-lead. The Ratio bottom can proceed both image acquisition (DNG and JPG format) and image ratio (YFP/CFP, only in DNG format) through smartphone camera and display in color map (the rainbow color plate is the predestined setting). Such kind of visualization can help users to easily judge whether the sample is contaminated with Pb.

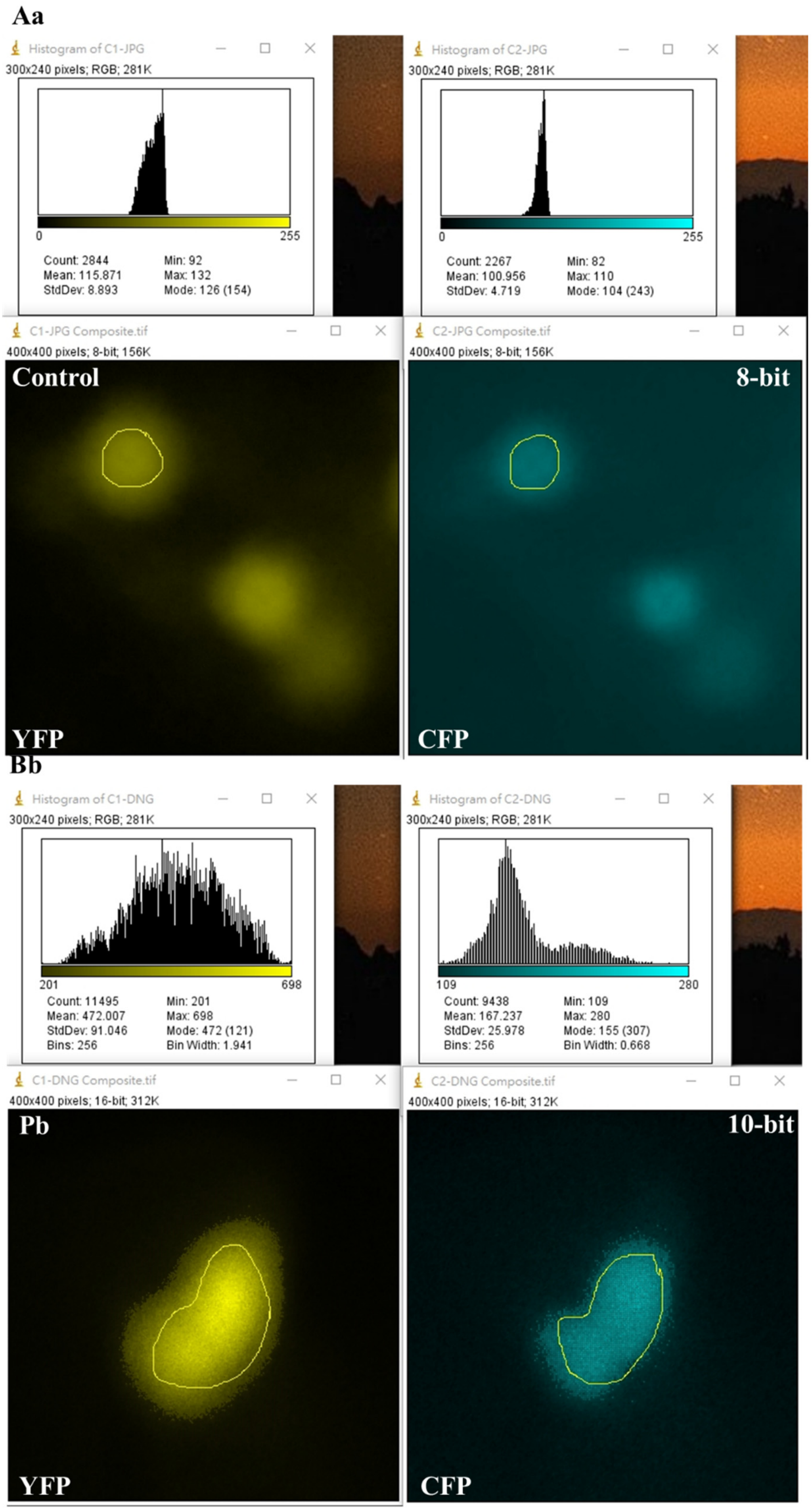
Aa
Histogram of C1-JPG
300x240 pixels; RGB; 281K
0
255
Count: 2844
Mean: 115.871
StdDev: 8.893
Min: 92
Max: 132
Mode: 126 (154)
Histogram of C2-JPG
300x240 pixels; RGB; 281K
0
255
Count: 2267
Mean: 100.956
StdDev: 4.719
Min: 82
Max: 110
Mode: 104 (243)
C1-JPG Composite.tif
400x400 pixels; 8-bit; 156K
Control
YFP
C2-JPG Composite.tif
400x400 pixels; 8-bit; 156K
8-bit
CFP
Bb
Histogram of C1-DNG
300x240 pixels; RGB; 281K
201
698
Count: 11495
Mean: 472.007
StdDev: 91.046
Bins: 256
Min: 201
Max: 698
Mode: 472 (121)
Bin Width: 1.941
Histogram of C2-DNG
300x240 pixels; RGB; 281K
109
280
Count: 9438
Mean: 167.237
StdDev: 25.978
Bins: 256
Min: 109
Max: 280
Mode: 155 (307)
Bin Width: 0.668
C1-DNG Composite.tif
400x400 pixels; 16-bit; 312K
Pb
YFP
C2-DNG Composite.tif
400x400 pixels; 16-bit; 312K
10-bit
CFP

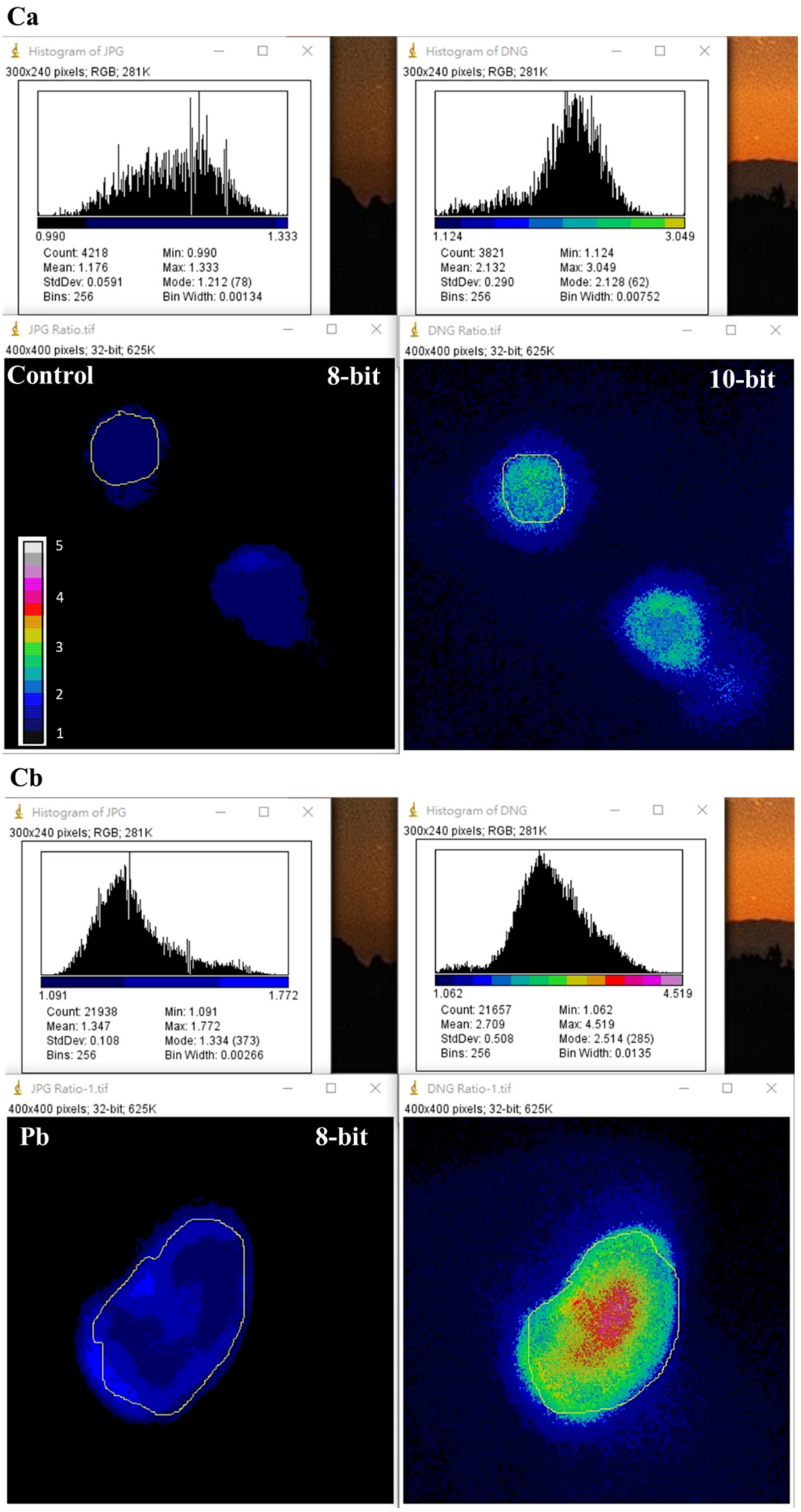
Ca
Histogram of JPG
300x240 pixels; RGB; 281K
0.990
1.333
Count: 4218
Mean: 1.176
StdDev: 0.0591
Bins: 256
Min: 0.990
Max: 1.333
Mode: 1.212 (78)
Bin Width: 0.00134
Histogram of DNG
300x240 pixels; RGB; 281K
1.124
3.049
Count: 3821
Mean: 2.132
StdDev: 0.290
Bins: 256
Min: 1.124
Max: 3.049
Mode: 2.128 (62)
Bin Width: 0.00752
JPG Ratio.tif
400x400 pixels; 32-bit; 625K
Control
8-bit
5
4
3
2
1
DNG Ratio.tif
400x400 pixels; 32-bit; 625K
10-bit
Cb
Histogram of JPG
300x240 pixels; RGB; 281K
1.091
1.772
Count: 21938
Mean: 1.347
StdDev: 0.108
Bins: 256
Min: 1.091
Max: 1.772
Mode: 1.334 (373)
Bin Width: 0.00266
Histogram of DNG
300x240 pixels; RGB; 281K
1.062
4.519
Count: 21657
Mean: 2.709
StdDev: 0.508
Bins: 256
Min: 1.062
Max: 4.519
Mode: 2.514 (285)
Bin Width: 0.0135
JPG Ratio-1.tif
400x400 pixels; 32-bit; 625K
Pb
8-bit
DNG Ratio-1.tif
400x400 pixels; 32-bit; 625K

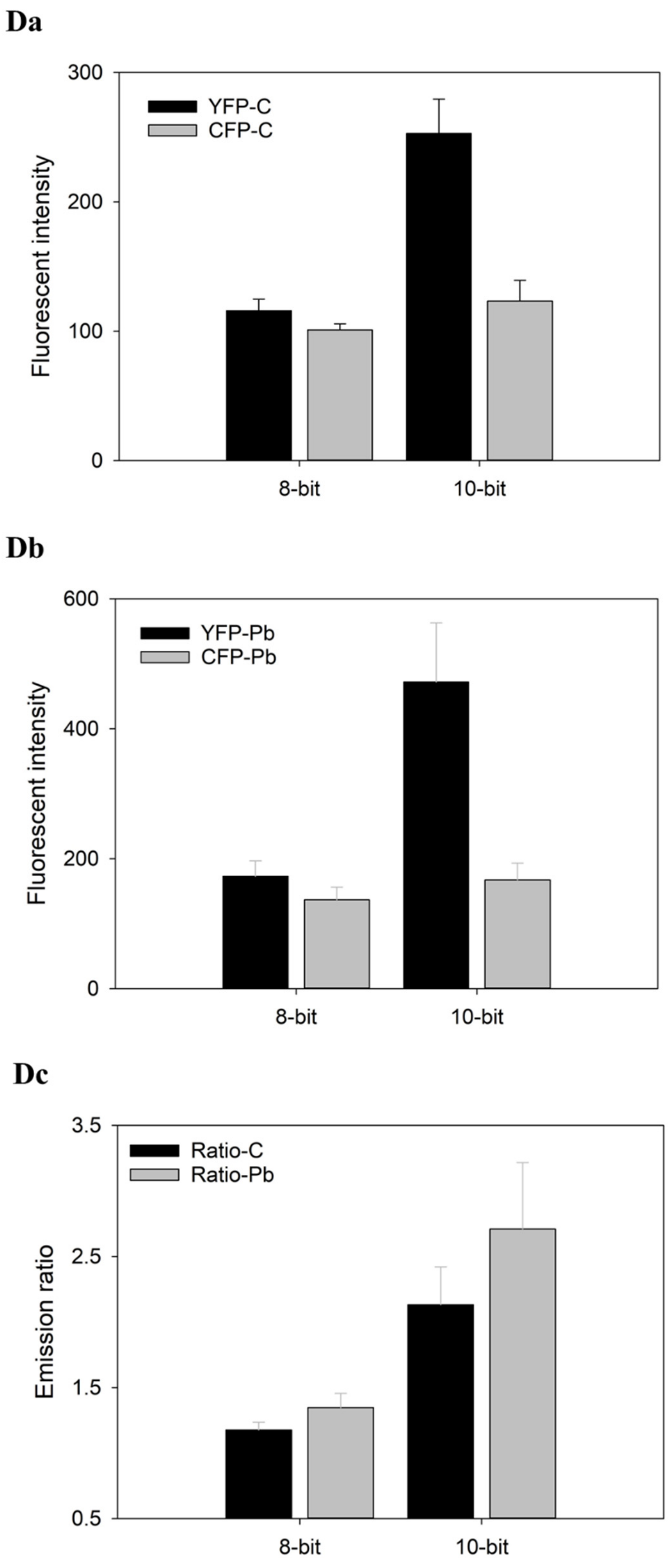
Da
300
200
100
0
Fluorescent intensity
YFP-C
CFP-C
8-bit
10-bit
Db
600
400
200
0
Fluorescent intensity
YFP-Pb
CFP-Pb
8-bit
10-bit
Dc
3.5
2.5
1.5
0.5
Emission ratio
Ratio-C
Ratio-Pb
8-bit
10-bit

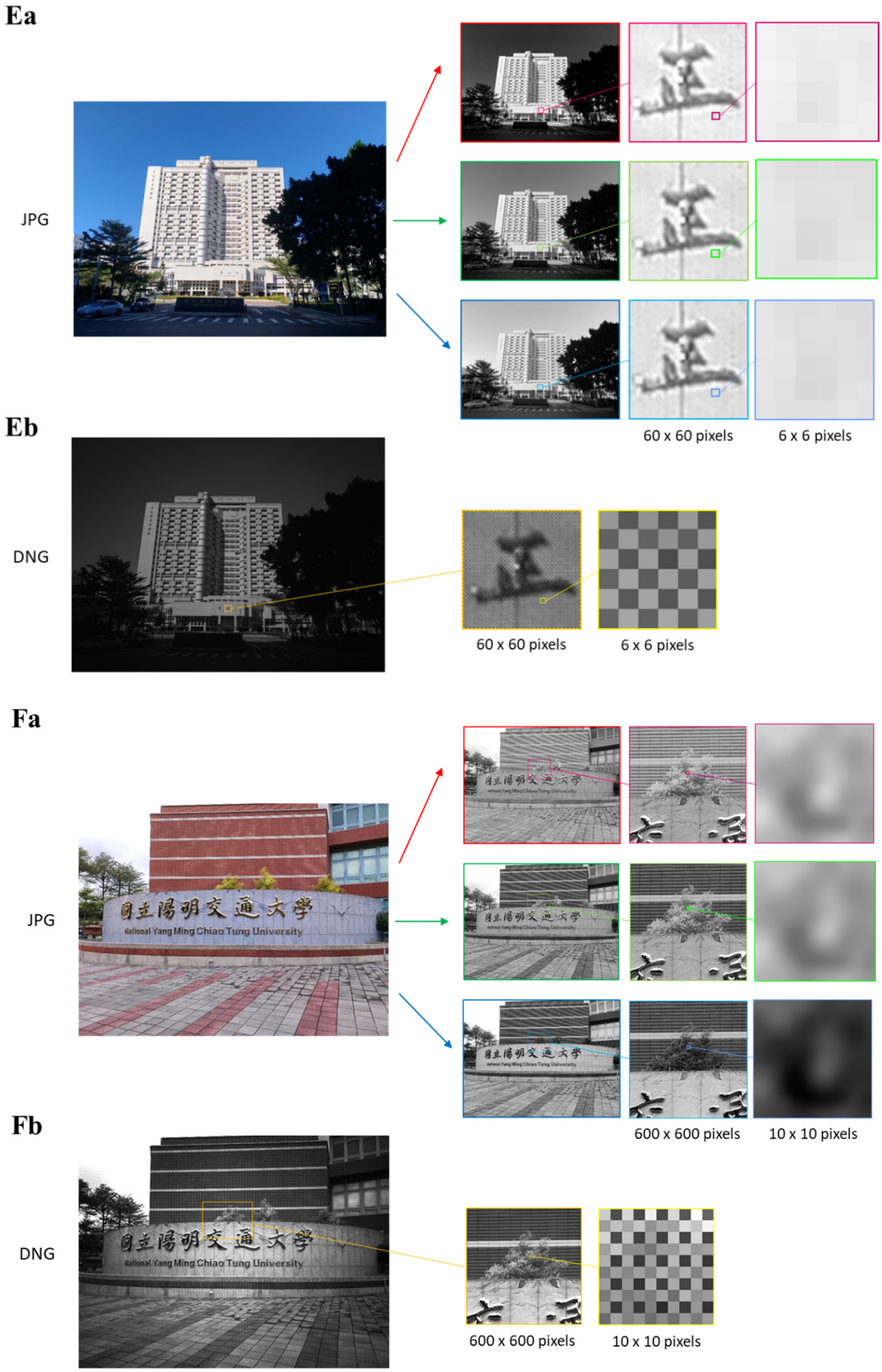

Figure S3. Image depth and image properties taken from smartphones. (**A**) Image depth at 8 bits in jpg format. (**B**) Image depth in 10 bits (DNG) format. (**C**) Ratio images original from 8 bits (**A**) or 10 bits (**B**) files. (**D**) Bar graphs from (**A**) to (**C**). (**E**,**F**)- The black spots observed as shown in Figure 3 are not due to pMet-lead.

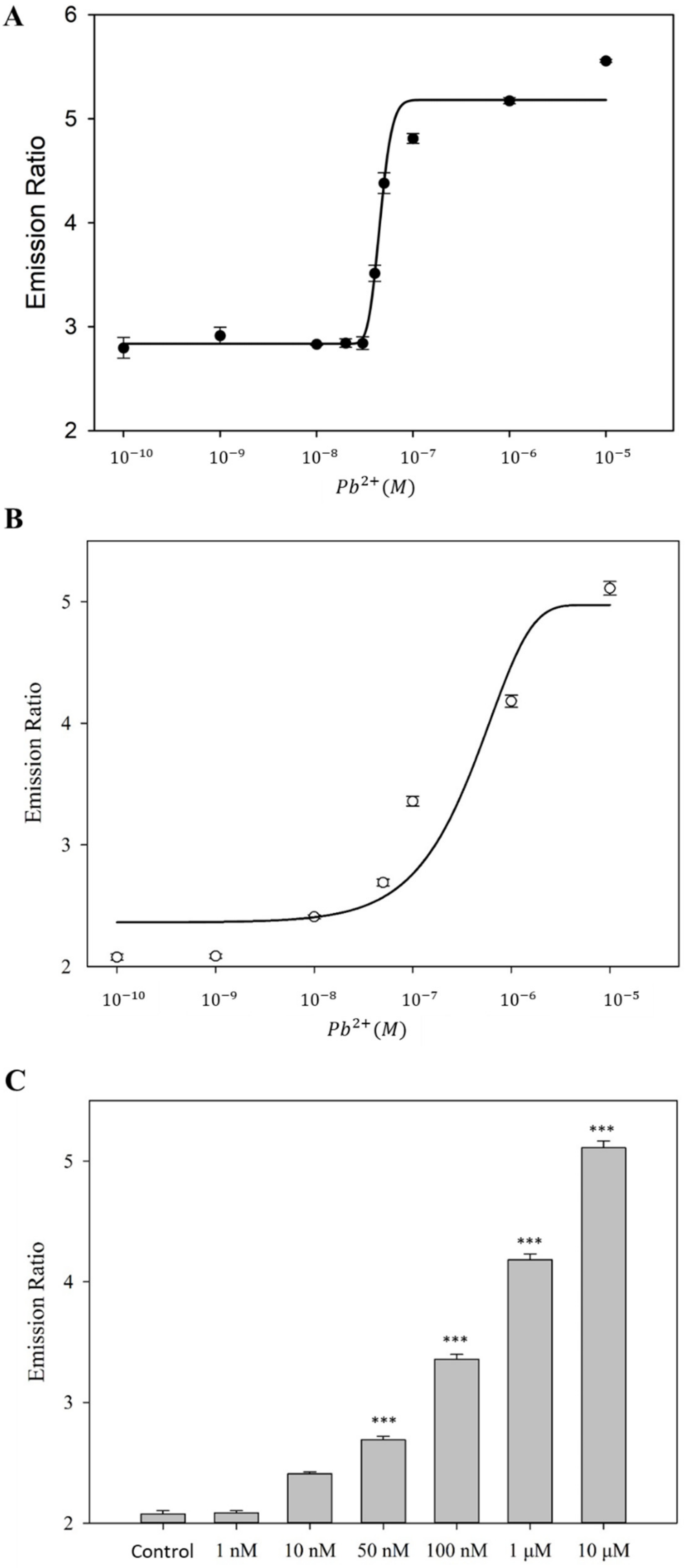

Figure S4. Sensing ability of pMet-lead and Met-lead 1.44 M1. (**A**,**B**) Titration experiments of Met-lead 1.44 M1 under pMet-lead (**A**) or under FRET ratio microscope (**B**). (**C**) Bar graphs of FRET ratio Pb sensing under various concentrations of Pb through FRET ration microscope by Met-lead 1.44 M1.

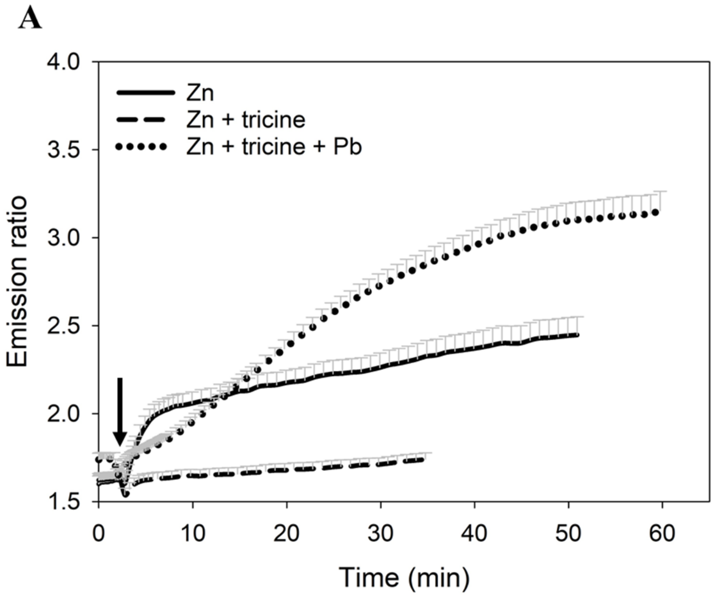

	ddH2O from MSL laboratory
Zn	11 μM added
Pb	10 μM added

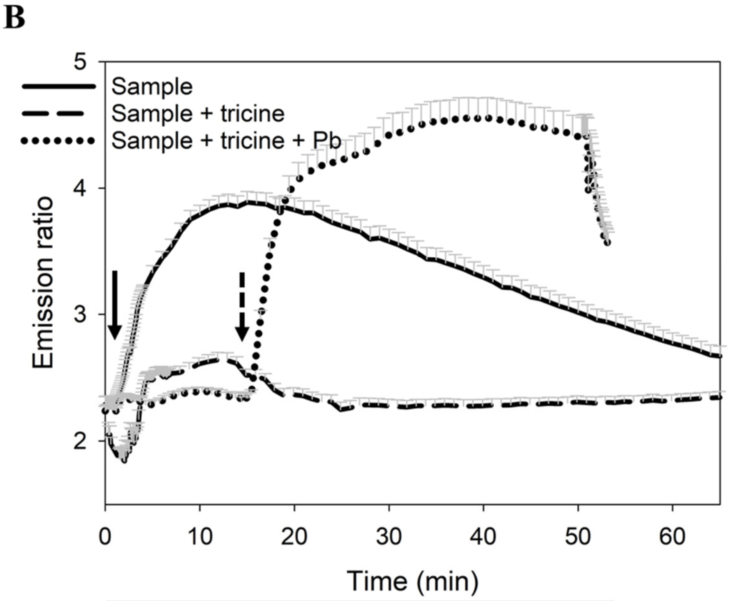

	Tap water from MSL laboratory
Zn	977.8 (ppb, μg/dL); 11 μM
Pb	1.95 (ppb, μg/dL); 9.75 nM

Figure S5. Full time scale of experiments shown in Figure 4. (**A**), Time-lapse changes in the Y/C ratio value under the recording of the Met-lead biosensing system. The double-distilled water from the laboratory was pre-mixed with certain concentrations of zinc alone (11 μM, Zn, in solid line), with additional tricine (10 mM, Zn + tricine, in dash line), or with additional tricine and Pb (10 μM, Zn + tricine + Pb, in dotted line) introduced at the time point as the arrows indicated. (**B**) Tap water from the faucet of the laboratory as the sample without (Sample, in solid line) or with tricine (10 mM) pre-mixed (Sample + tricine, in dash line) or with additional Pb (10 μM, Zn + tricine + Pb, in dotted line) to be added into the Met-lead biosensing system as the arrow indicates. All the experimental sample tests were with ionomycin (5 μM).

Additional Movies.

Movie S1. Time-lapse FRET ratio color data of Met-lead 1.44 M1 under FRET ratio microscope on pure water when adding certain amounts of zinc (11 μM). The ratio color bar is from 1.1 to 3.5.

Movie S2. Time-lapse FRET ratio color data of Met-lead 1.44 M1 under FRET ratio microscope on pure water when adding certain amounts of zinc (11 μM) pre-incubated with zinc chelator tricine (10 mM). The ratio color bar is from 1.1 to 4.0.

Movie S3. Time-lapse FRET ratio color data of Met-lead 1.44 M1 under FRET ratio microscope on pure water when adding certain amounts of zinc (11 μM) pre-incubated with zinc chelator tricine (10 mM) and additional treatment with Pb (10 μM). The ratio color bar is from 1.1 to 3.5.

Movie S4. Time-lapse FRET ratio color data of Met-lead 1.44 M1 under FRET ratio microscope on real water sample (tap from laboratory). The ratio color bar is from 1.5 to 3.5.

Movie S5. Time-lapse FRET ratio color data of Met-lead 1.44 M1 under FRET ratio microscope on real water sample (tap from laboratory) pre-incubated with zinc chelator tricine (10 mM). The ratio color bar is from 1.5 to 3.5.

Movie S6. Time-lapse FRET ratio color data of Met-lead 1.44 M1 under FRET ratio microscope on real water sample (tap from laboratory) pre-incubated with zinc chelator tricine (10 mM) and additional treatment with Pb (10 μM). The ratio color bar is from 1.5 to 6.0.

Movie S7. The animated flowchart of the iMet-lead app for smartphones integrated with the device pMet-lead.
