# Supplementary figures and images for: Portable FRET-Based Biosensor Device for On-Site Lead Detection
Source: Biosensors (Basel). 2022 Mar 2;12(3):157. doi: 10.3390/bios12030157 (PMC8946079; doi:10.3390/bios12030157)

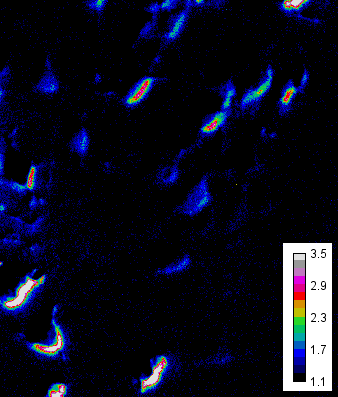

Supplement: Supplementary file 1 [file biosensors-12-00157-s001.zip › biosensors-1592399-supplementary/Movie S1.gif]

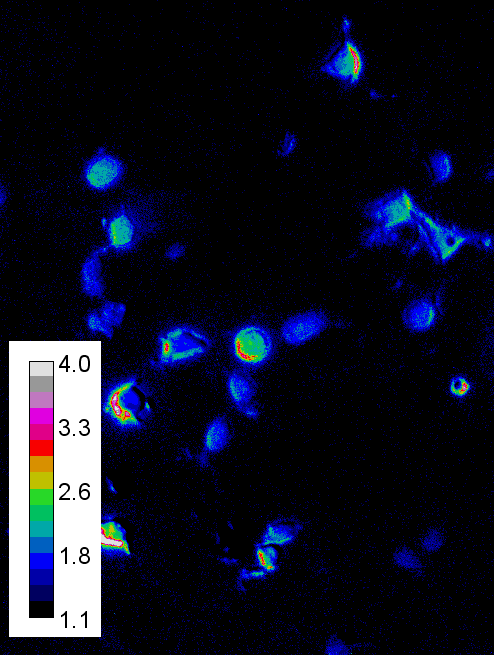

Supplement: Supplementary file 1 [file biosensors-12-00157-s001.zip › biosensors-1592399-supplementary/Movie S2.gif]

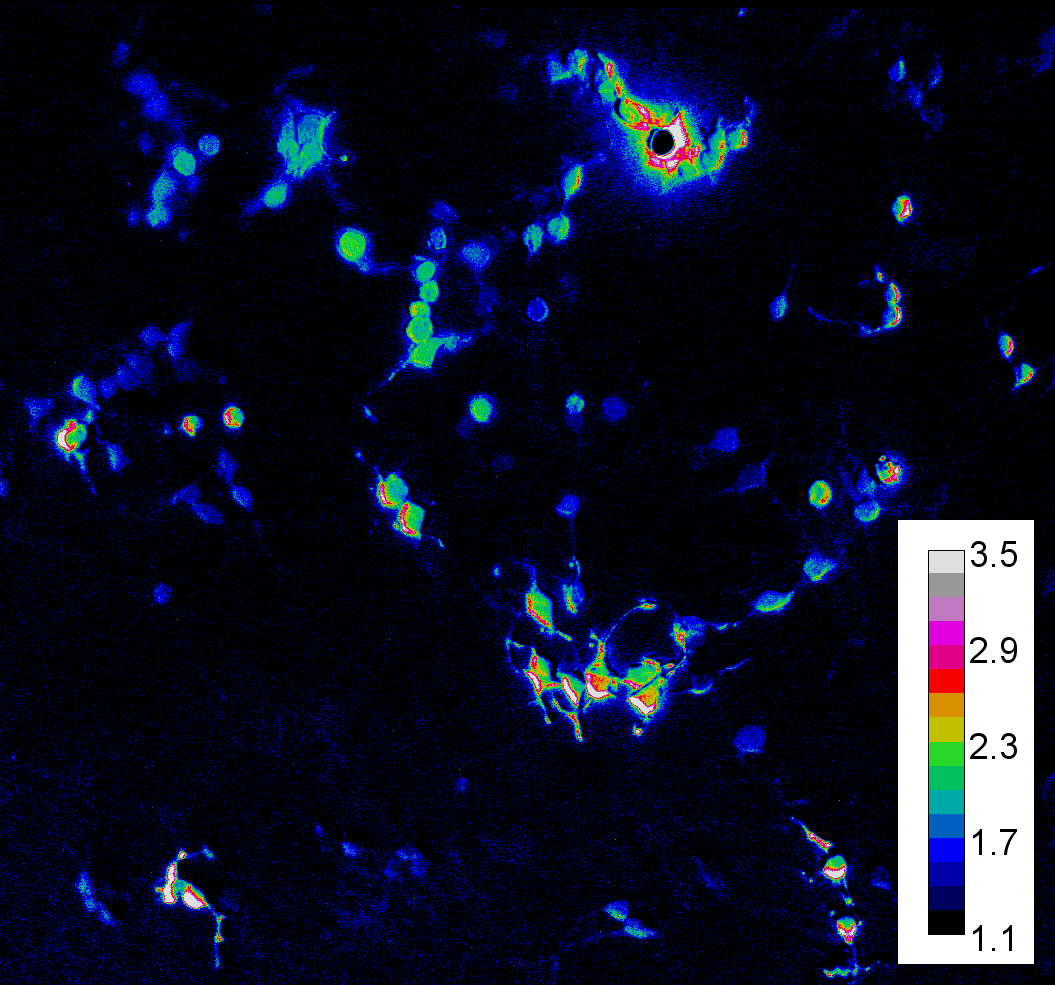

Supplement: Supplementary file 1 [file biosensors-12-00157-s001.zip › biosensors-1592399-supplementary/Movie S3.gif]

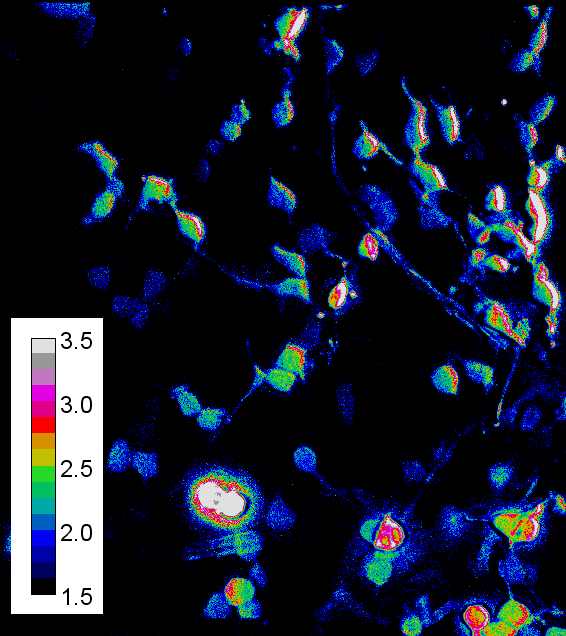

Supplement: Supplementary file 1 [file biosensors-12-00157-s001.zip › biosensors-1592399-supplementary/Movie S4.gif]

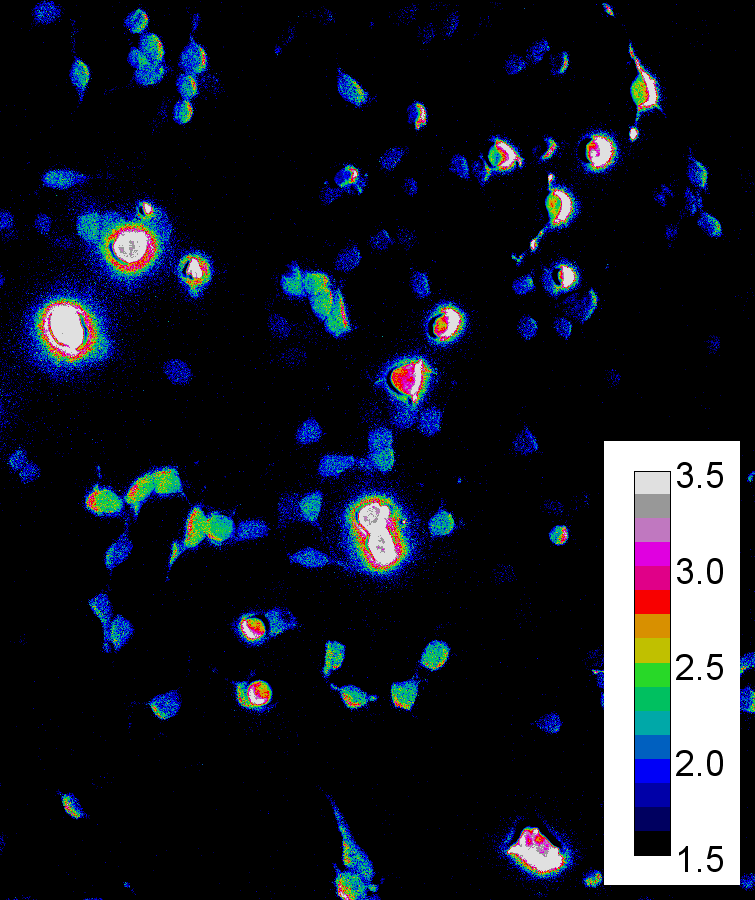

Supplement: Supplementary file 1 [file biosensors-12-00157-s001.zip › biosensors-1592399-supplementary/Movie S5.gif]

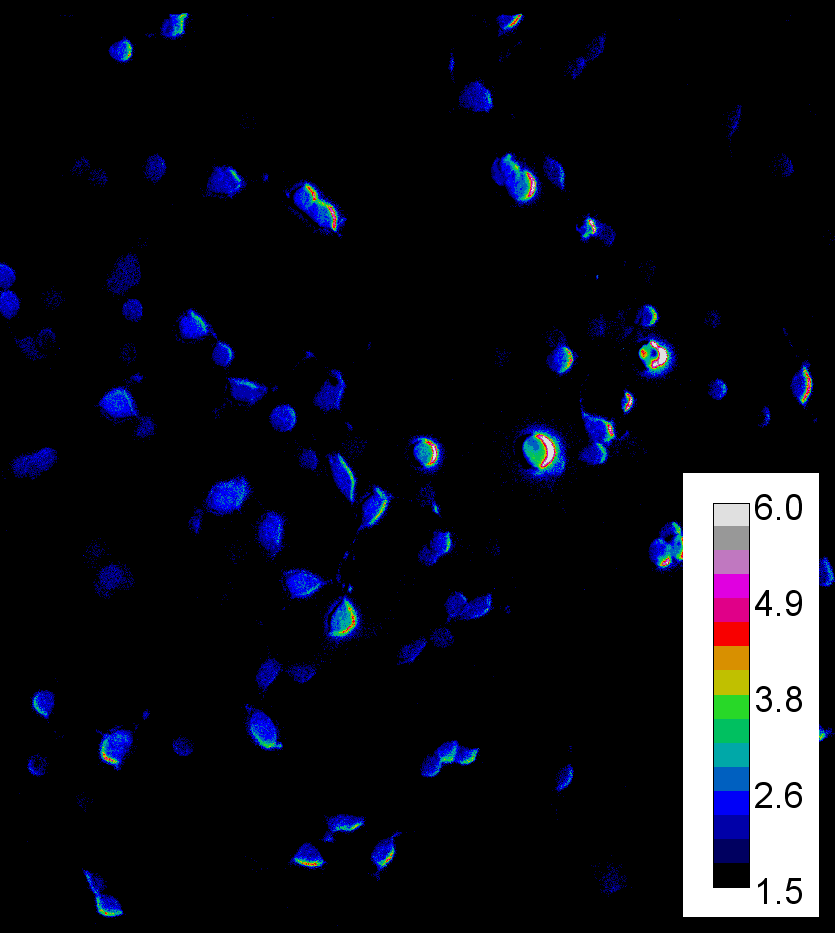

Supplement: Supplementary file 1 [file biosensors-12-00157-s001.zip › biosensors-1592399-supplementary/Movie S6.gif]
